# Retraction Note: Targeting KDM4A-AS1 represses AR/AR-Vs deubiquitination and enhances enzalutamide response in CRPC

**DOI:** 10.1038/s41388-023-02790-8

**Published:** 2023-07-31

**Authors:** Boya Zhang, Mingpeng Zhang, Yanjie Yang, Qi Li, Jianpeng Yu, Shimiao Zhu, Yuanjie Niu, Zhiqun Shang

**Affiliations:** Tianjin Institute of Urology, The Second Hospital of Tianjin Medical University, Tianjin, 300211 China

**Keywords:** Cancer therapeutic resistance, Prostate cancer

Retraction to: *Oncogene* 10.1038/s41388-021-02103-x, published online 10 November 2021

The Editors-in-Chief have retracted this article because concerns have been raised regarding integrity of the images presented here. Specifically,

- There appears to be overlap between the top left and top right panels of Fig. 2E.
- There appears to be overlap between the top three panels of Fig. 2F. The bottom left and bottom right panels of Fig. 2F appear to be the same.
- C4-2 blot in Fig. 3E appears to be the same as blot presented as LNCaP-AI in Fig. 3I.
- There appears to be a partial overlap between two panels of Fig. 4J which are described as representing different experimental conditions.
- Part of Fig. 5E (GAPDH blot) appears to have previously been published as part of Fig. 5E in [1] by some of the same authors.

The Editors-in-Chief therefore no longer have confidence in the reliability of the data presented in this article.

Zhiqun Shang does not agree to this retraction. Boya Zhang has not explicitly stated whether they agree to this retraction. Mingpeng Zhang, Qi Li, Jianpeng Yu, and Yuanjie Niu have not responded to correspondence about this retraction. The Publisher has not been able to obtain a current email address for Yanjie Yang or Shimiao Zhu.

## References

[1] Zhang B, Zhang M, Shen C (2021). LncRNA PCBP1-AS1-mediated AR/AR-V7 deubiquitination enhances prostate cancer enzalutamide resistance. Cell Death Dis.

